# Ectopic variceal bleeding after hepatobiliary surgery: A case report

**DOI:** 10.1097/MD.0000000000024975

**Published:** 2021-03-05

**Authors:** Yang Tai, Huan Tong, Bo Wei, Hao Wu

**Affiliations:** a Department of Gastroenterology; b Laboratory of Gastroenterology and Hepatology, West China Hospital, Sichuan University, Chengdu, China.

**Keywords:** hepatobiliary surgery, jejunal varices, portal hypertension, portal venous stenosis

## Abstract

**Rationale:**

Jejunal varices are rare in portal hypertension and are often difficult to diagnose and treat. Herein, we present a case of gastrointestinal bleeding due to jejunal varices after hepatobiliary surgery.

**Patient concerns:**

A 69-year-old man presented with recurrent massive gastrointestinal bleeding. He underwent partial right hepatectomy and cholangiojejunostomy 2 years prior to the first onset of bleeding. Two sessions of endoscopic vessel ligation for esophageal varices were performed afterwards, and hematemesis resolved completely, but massive melena still recurred during the following 5 years.

**Diagnosis:**

The patient was diagnosed with jejunal varices caused by portal venous stenosis after hepatobiliary surgery.

**Intervention:**

Portal venous angioplasty using balloon dilation and stent implantation was performed.

**Outcomes:**

After the intervention procedure, the patient did not experience any onset of gastrointestinal bleeding during follow-up.

**Lessons:**

Hepatopancreatobiliary could lead to the formation of jejunal varices. The combined use of capsule endoscopy, contrast-enhanced computed tomography, and sometimes portal venography is a promising strategy to search for jejunal varices. Transcatheter angioplasty appears to be a safe and effective method for treatment of jejunal varices in certain appropriate cases.

## 1 Introduction

Ectopic varices are a rare and often neglected cause of gastrointestinal bleeding in patients with portal hypertension. The jejunum and ileum account for 17% of the bleeding sites of ectopic varices.^[1]^ Unlike esophagogastric varices, intestinal varices are usually difficult to access using conventional endoscopy.^[2]^ Although varices are usually found in liver cirrhosis, they can develop in non-cirrhotic conditions.^[3]^ The uncommon scenario of intestinal varices without liver cirrhosis tests the clinical mindset and emergent response of practitioners. Benign portal venous stenosis is a common complication after hepatopancreatobiliary surgery, resulting in increased portal pressure and varices formation in the esophagogastric sites and intestine.^[4]^ Herein, we present a case of jejunal variceal bleeding after hepaticbiliary surgery, with the aim of raising awareness of gastrointestinal bleeding with this atypical and rare etiology.

## 2 Case presentation

**Figure 1:**
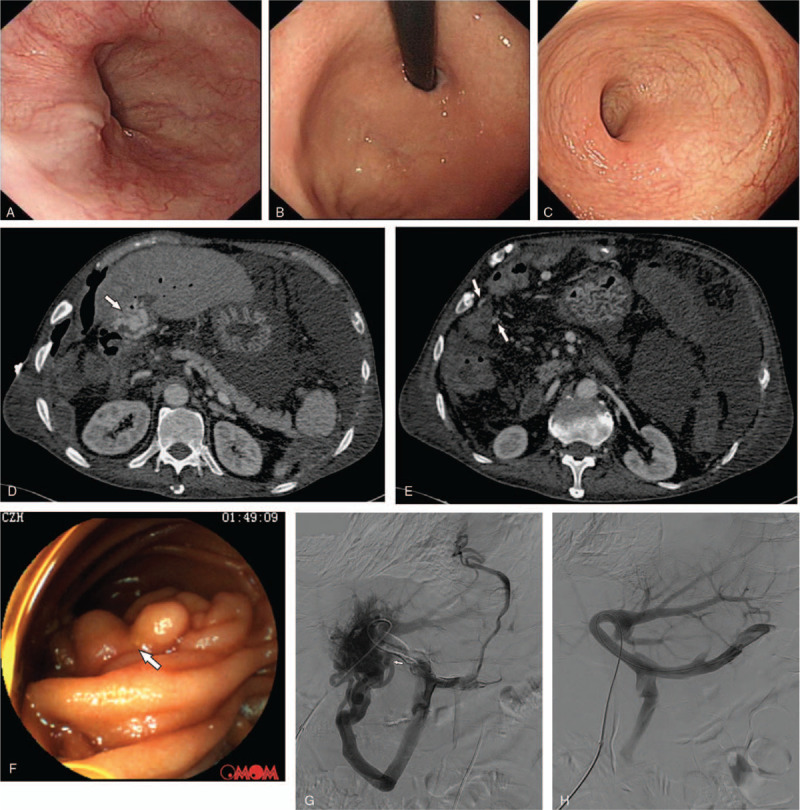
Normal appearances of esophagus (A), stomach fundus (B), and rectum (C) are shown by esophagogastroduodenoscopy and colonoscopy, respectively. Portal cavernoma and dilated veins accompanies with the small intestine are shown by white arrows in (D) and (E), respectively. Ectopic varices in jejunum are shown by white arrow under capsule endoscopy (F). Portal venograpy imaging before and after portal venous angioplasty are shown in (G) and (H), respectively. The white arrow in (G) indicates jejunal varices.

A 69-year-old man presented with recurrent melena that had persisted for 5 years. He underwent partial right hepatobectomy and cholangiojejunostomy due to hepatolithiasis and hilar biliary stricture 7 years prior. The first onset of massive melena with hematemesis occurred 2 years after surgery. Two sessions of endoscopic vessel ligation for esophageal varices were performed afterwards, and hematemesis resolved completely, but massive melena still recurred during the following 5 years. He had no history of alcohol or drug intake. At admission, the physical examination was unremarkable, and laboratory tests showed decreased hemoglobin (65 g/L, normal value [nv]: 130–175 g/L), normal leukocyte (6.67 × 10^9^/L, nv: 3.5–9.5 × 10^9^/L) and platelet (110 × 10^9^/L, nv: 100–300 × 10^9^/L) counts, normal total bilirubin (23.9 μmol/L, nv: 5.0–28.0 μmol/L), and aminotransferases (alanine aminotransaminase 10 IU/L, nv: < 50 IU/L; aspartate aminotransferase 7 IU/L, nv: < 40 IU/L), decreased albumin (24.8 g/L, nv: 40–55 g/L), and increased prothrombin time (23.0 seconds, nv: 9.6–12.8 seconds). Neither esophagogastroduodenoscopy nor colonoscopy revealed varices or other lesions (Fig. 1A-C). However, abdominal contrast-enhanced computed tomography (CECT) suggested portal cavernoma and dilated veins adjoining the small intestine (Fig. 1D, E). Capsule endoscopy (OMOM, Jinshan Science & Technology, China) screened the entire small intestine and found jejunal varices without active bleeding (Fig. 1F). Additionally, transient elastography showed a normal range of liver stiffness measurements.

Considering the coexistence of portal cavernoma and jejunal varices, portal venography via percutaneous transhepatic access was performed, showing that the portal vein underwent severe stenosis to be nearly occluded at the hepatic hilum, and the superior mesenteric vein (SMV) distributed abundant varices to encompass the hepatic hilum (Fig. 1G). Portal venous angioplasty by balloon dilation and stent implantation under X-ray was performed thereafter, with a superior mesenteric-systemic pressure gradient decrement from 16 mm Hg to 7 mm Hg. In addition, the portal vein at the hepatic hilum was clearly displayed, and varices from the SMV could not be observed after stent implantation (Fig. 1H). During the follow-up of 3 years, he did not experience any onset of gastrointestinal bleeding, and hemoglobin, albumin, and prothrombin time gradually recovered to normal levels.

## 3 Discussion

Portosystemic collateral vessel formation is one of the most common complications of portal hypertension, which increases the risk of variceal bleeding and subsequent death.^[5]^ Although the majority of varices are present at the gastroesophageal junction and stomach fundus, few cases still develop varices at extra-esophagogastric sites, which are known as ectopic varices.^[6]^ Theoretically, ectopic varices could develop in the area between the portal and systemic venous systems, including the duodenum, jejunum, ileum, colon, and rectum.^[7]^

As the majority of intestinal varices reside beyond the screening capacity of conventional endoscopy, they are difficult to confirm on most occasions. Capsule endoscopy and enteroscopy are recommended as supplements for ectopic varices navigation.^[8]^ However, it is noted that endoscopy is incompetent in searching for any vascular abnormality beyond the alimentary tract, whereas CECT and digital subtraction angiography perform better on such issues. In particular, portal venography is crucial for varices visualization.^[9]^ Although jejunal varices are only confirmed by exploratory laparotomy under certain extreme circumstances, systemic and careful workup is still needed to search for varices. This case involved the use of a combination of capsule endoscopy, CECT, and portal venography, displaying the varices and obtaining enough information for the successive procedure.

Although most ectopic varices develop in liver cirrhosis, non-cirrhotic conditions should not be downplayed. Minowa et al summarized cases of ideal variceal bleeding reported between 1982 and 2009. Five out of 21 patients had liver cirrhosis, and among those 5 non-cirrhotic cases, only 3 patients underwent abdominal surgery (1 hysterectomy, 1 for ectopic pregnancy, and 1 for acute appendicitis).^[10]^ As for jejunal varices, liver cirrhosis is still the leading etiology. However, surgery should not be depreciated while considering the probable etiology of jejunal varices. There are still limited case reports on jejunal varices without liver cirrhosis that developed after abdominal surgery.^[11–13]^ Unlike surgery involving the gall bladder, pancreas, and duodenum, this patient underwent surgery mainly in the liver, which would affect the portal vein thereafter, leading to jejunal varices and urgent bleeding. Portal venous stenosis may occur after hepatic surgery due to portal venous reconstruction and postoperative inflammation, which impedes blood flow into the liver and leads to portal hypertension.^[14]^

Treatment options for intestinal varices include surgery, enteroscopic sclerotherapy, transjugular intrahepatic portal systemic shunt, transcatheter arterial embolization, and portal venous dilatation with or without stent placement.^[15–17]^ The etiology of varices, portal venous condition, procedure efficacy, and comorbidity should be taken into consideration during treatment choice making. In this case, jejunal varices are associated with past hepaticbiliary surgery; therefore, it would not be the first choice to deploy the second surgery to tackle the varices. In addition, endoscopic therapy is not easily accessible because of the troublesome location of varices. Transcatheter angioplasty (balloon dilation plus stent implantation) seems to be a relatively safe and effective treatment to reconstruct the portal vein to mitigate venous stenosis and varices, and extra stent implantation adds a better long-term patency and lower recurrence rate compared to balloon dilatation alone.^[18]^

In conclusion, jejunal varices are rare and difficult to diagnose. Past history of hepatopancreatobiliary should raise the alert of jejunal varices in gastrointestinal bleeding individuals with unconfirmed diagnosis. The combined use of capsule endoscopy, CECT, and sometimes portal venography is a promising strategy to search for jejunal varices. Transcatheter angioplasty appears to be a safe and effective method for treatment of jejunal varices in certain appropriate cases.

## Author contributions

**Conceptualization:** Hao Wu.

**Data curation:** Yang Tai, Huan Tong, Bo Wei.

**Funding acquisition:** Yang Tai, Huan Tong, Hao Wu.

**Supervision:** Hao Wu.

**Writing – original draft:** Yang Tai, Huan Tong.

**Writing – review & editing:** Huan Tong, Hao Wu.
